# Predicting outcomes in autoimmune encephalitis: Will scales and scores ever be enough?

**DOI:** 10.1111/dmcn.70463

**Published:** 2026-08-20

**Authors:** Yoshua Collins-Sawaragi, Sukhvir Wright

**Affiliations:** 1 Faculty of Life Sciences & Medicine, King’s College London, London, UK; 2 Institute of Health and Neurodevelopment, Aston University, Birmingham, UK; 3 Department of Neurology, Birmingham Children’s Hospital, Birmingham, UK

Worldwide, one person every 20 seconds is diagnosed with encephalitis (http://encephalitis.info/flames/). Autoimmune aetiologies are as common as infectious causes and detection is increasing. While we are succeeding in early diagnosis, initiation of immunotherapy, and preventing relapses, outcomes in autoimmune encephalitis remain frustratingly static. Children are disproportionally affected; globally, encephalitis is the fourth leading cause of neurological health loss (measured in disability-adjusted life years) in children under 5 years of age.^1^

The modified Rankin Scale (mRS) has been utilized widely as an outcome measure in paediatric and adult autoimmune encephalitis; but as a scale initially designed for adult stroke patients, with emphasis on motor outcomes, it does not capture the complex symptomatology of autoimmune encephalitis. This has led to the development of specific scales, for example the anti-NMDAR-encephalitis (NMDARE) 1-year functional status (NEOS)^2^ and the Clinical Assessment Scale in Autoimmune Encephalitis (CASE).^3^ CASE was developed for adults but has since been tested prospectively in children,^4^ while a modified version (Ped-CASE) incorporating the child’s development status was tested in children with seronegative autoimmune encephalitis.^5^ Both were able to monitor severity and predict functional outcomes more accurately than mRS or functional status scores.

Madaan et al. present a validation study on the Paediatric Autoimmune Encephalitis Severity Scale (PASS) created specifically for children with autoimmune encephalitis by an expert consensus group to enable monitoring of disease progress and outcomes.^6^ Longitudinal data on a prospective cohort is presented and compared to CASE and mRS. The authors show raters scored patients in a similar fashion on PASS, and the highest PASS score in the acute period (indicating a higher disease severity) correlated with worse long-term quality of life outcome measures. As seen in this study, engagement of patients, families, and charity stakeholders is a welcomed step in ensuring inclusion of measures that matter to them, something which is often missed in purely clinical driven efforts. However, an inherent problem with validating any new scoring system in a rare disease is sample size (*n* = 27 in Madaan et al.), therefore care should be taken not to assume superiority of one over another.

The question remains whether potentially subjective scores will ever be sufficient alone to accurately measure disease severity and predict outcomes. Incorporating neurophysiological, neuroimaging, and biological markers through machine learning will likely lead to the creation of advanced predictive models and these are already emerging in adult NMDARE,^7^ and other neuroinflammatory diseases.

Where scoring systems do come in useful is creating a common language between clinicians. Moving forwards, further consensus will be required to adopt a universal scoring system to facilitate standardized outcome measurements. The current practice of using different outcome measures makes data interpretation and comparison challenging.

This need is even more pressing as children are now being included in autoimmune encephalitis clinical trials, for example ExTINGUISH investigating the role of intravenous inebilizumab in NMDARE.^8^ As our understanding of auto-immune encephalitis and treatments advances at pace, we do not want the inclusion of children and young people to be left behind.

## Data Availability

Not required.
